# Determination of indoxyl sulfate by spectrofluorimetric method in human plasma through extraction with deep eutectic solvent

**DOI:** 10.1186/s13065-024-01172-9

**Published:** 2024-03-30

**Authors:** Samira Shafiee, Siavoush Dastmalchi, Afshin Gharekhani, Ali Shayanfar

**Affiliations:** 1 Student Research Committee, Tabriz University of Medical Sciences, Tabriz, Iran; 2 Biotechnology Research Center, Tabriz University of Medical Sciences, Tabriz, Iran; 3 Faculty of Pharmacy, Tabriz University of Medical Sciences, Tabriz, Iran; 4 Faculty of Pharmacy, Near East University, Mersin 10, Nicosia, POBOX: 99138 North Cyprus Turkey; 5 Pharmaceutical Analysis Research Center, Tabriz University of Medical Sciences, Tabriz, Iran

**Keywords:** Deep eutectic solvent (DES), Extraction, Indoxyl sulfate (IS), Spectrofluorimetry

## Abstract

A rapid and efficient analytical method was established to quantify indoxyl sulfate (IS) in plasma through extraction technique with a deep eutectic solvent (DES) and spectrofluorimetric method. DES (choline chloride: urea) was mixed with plasma samples for the extraction of IS, followed by the addition of dipotassium hydrogen phosphate (K_2_HPO_4_) solution to form an aqueous two-phase system. The fluorescence intensity of IS which was first extracted to the DES-rich-phase and then back-extracted into the salt-rich-phase, was measured by spectrofluorimetric method. Some key factors such as pH, centrifugation speed and time, the volume ratio of DES/salt, and salt concentration were optimized. Under the optimized conditions, the suggested method had a dynamic range between 20 and 160 µg/mL with a coefficient of determination (R^2^) of 0.99. Precision (relative standard deviation) was less than 15% and accuracy (% relative recovery) was ± 15% at the nominal concentration level. In addition, results showed that IS levels in real samples were higher than 40 µg/mL which was compatible with reported IS levels in end-stage renal disease (ESRD) patients. Overall, all the results reflect the fact that the presented analytical method can potentially be used for the determination of IS in real plasma samples.

**Supplementary Information:**

The online version contains supplementary material available at 10.1186/s13065-024-01172-9.

## Introduction

Chronic kidney disease (CKD) is an increasingly widespread disorder with a high incidence and fatality rate worldwide [1]. It is a general term that includes all chronic kidney disorders from minimally impaired kidney function to end-stage renal disease (ESRD) [2]. Chemicals that are typically eliminated by the kidney are known as uremic toxins and accumulate in blood as CKD progresses [3]. Protein-bound uremic toxins have a high binding affinity, making dialysis a challenging kidney replacement therapy for their removal/elimination in CKD patients [4]. The uremic toxins retained during renal failure are deleterious to almost every organ and system in the body [5]. Indoxyl sulfate (IS), as a protein-bound uremic toxin, plays a key role in the progression of CKD due to its nephrotoxicity [6, 7], as well as CKD-associated complications including cardiovascular toxicity [8–11], bone toxicity [12], and anemia [13–15].

Measuring the concentration of IS and other uremic toxins during the evaluation of renal disorder could be utilized as a reliable biomarker for promoting timely individual medical therapies required to preserve kidney function [16]. Therefore, reliable analytical methods for quantifying uremic toxins are inevitably necessary. To measure various uremic toxins, including IS, a variety of analytical techniques, especially those based on chromatography, like liquid chromatography (LC) have been frequently employed. LC is among the most commonly used analytical methods for determining the level of uremic toxins in biofluids [17]. Due to the native fluorescence property of IS, a detector has been utilized for the analysis of IS in biological samples in numerous studies [18–23].

Table 1 shows a list of some available methods addressing the analytical features used for measuring IS in biofluids. Techniques based on chromatography are time-consuming, laborious, and the related professional apparatus for analysis is of high-price. Owing to these shortcomings, the reference standard LC-MS and LC-MS/MS methodologies are incompatible with routine clinical research and real-time on-site analysis [24, 25]. Therefore, generating a rapid, sensitive, and precise technique is needed for measuring IS in plasma matrix. In this regard, fluorescence spectroscopy provides a rapid, simple, accurate and cost-effective approach to quantify fluorophore analytes [26]. Norouzi et al. [27] and Holmar et al. [28] proposed fluorescence spectroscopy as a feasible method to measure IS level in CKD patients and the removal ratio of IS, respectively. With regard to the required time and complexity of separating the targeted analyte from different matrices, sample preparation is the most crucial and challenging step in the analytical procedures [29]. However, extraction methodologies should be coupled to spectrofluorimetry to preserve acceptable analytical results as well as offering better options for conventional sample preparation [30].

**Table 1:** Some of the reported analytical methods for the determination of IS

Analytical method	Sample matrix	Linear range (µg/mL)	Ref.
LC–MS/MS	Human and animals’ plasma	0.1–100	[53]
LC–MS/MS	Human serum	0.5–10	[65]
LC–MS/MS	Human urine	0.1-106.5	[54]
LC–MS/MS	Human saliva	1500-200000	[56]
LC–MS/MS	Human serum, urine, and cell culture	1.06–8.52	[66]
LC-ESI-MS/MS	Human plasma	0.01-10	[67]
LC–MS/MS	Human serum and gastric juice	0.02–4.26	[61]
UHPLC–MS/MS	Human urine, plasma, and serum	0.019–9.860	[68]
Electrochemical	Rat serum	0.02–0.08	[50]
Spectrofluorimetry	Human plasma	2.5–40	[27]
Spectrophotometry	Human serum	1.59–20.2	[59]

LC-MS/MS: Liquid Chromatography with tandem mass spectrometry, UPLC–MS/MS: Ultra Performance Liquid Chromatography with tandem mass spectrometry, LC-ESI-MS/MS: Liquid Chromatography Electrospray Ionization Tandem Mass spectrometry

Organic solvents such as acetonitrile have been extensively employed in pharmaceutical and other industries for diverse applications including extraction, separation, and reaction. Remarkable extraction ability has been observed using organic solvents for both hydrophilic and hydrophobic molecules [31]. Despite the great effectiveness of organic solvents, they bear disadvantages including toxic properties, high inflammability, and non-biodegradability which are cause of long-term environmental and human toxicity concerns. As a result, numerous studies have concentrated on advancing to more environmentally safe solvents that are less toxic and biodegradable ones. Among several new solvents, deep eutectic solvents (DESs) have attracted the researchers interest in different fields due to their outstanding features including negligible environment and human toxicity, and have been also considered as “green solvents“ [32]. DESs are prepared as a result of combining two or more substances especially combinations of a hydrogen bond donors (HBDs) and acceptors (HBAs) with low melting points [33]. Besides, it has been discovered that DESs are useful tools for extracting various components [34] such as drugs, carbohydrates, proteins, and lipids [32].

The utilization of liquid-liquid extraction through DESs has been widely adopted due to the interesting physicochemical features, simple synthesis, and environmentally friendly properties compared with toxic organic solvents [30].

Moreover, aqueous two-phase system (ATPS) as a specific liquid-liquid extraction platform/approach, that involves extracting the solute from one phase to another, has been lately used in different areas including pharmaceuticals and biomarkers [35], enzymes, proteins, antibodies, nucleic acids, and antibiotics [36]. In this work, we present a simple and rapid analytical method with a focus on ATPS extraction by DES based on fluorescence property of IS to measure this biomarker in ESRD patients.

## Methods and materials

### Reagents

IS potassium salt was obtained from Sigma-Aldrich (USA). Dipotassium phosphate (K_2_HPO_4_), hydrochloric acid (37%), sodium hydroxide (NaOH), trichloroacetic acid (TCA), choline chloride, and urea were purchased from Merck (Darmstadt, Germany), and lab-made double distilled water was supplied for the preparation of solution.

### Apparatus and solutions

An oven (Tehran, Iran) was used for drying of choline chloride (ChCl). DES was prepared on Lab Companion heater with magnetic stirrer (HP-3000). The solutions were mixed using a Labtron (LS-100) vortex shaker (Tehran, Iran). In order to accelerate phase separation, a SIGMA centrifuge Model 1–14 (Osterode, Germany) was applied. Fluorescence spectra and intensity measurements were performed utilizing a 10-mm quartz cell and a Jasco FP-750 spectrofluorimeter (Kyoto, Japan) supplied with a 150-W xenon lamp. A stock solution of IS (2000 mg.mL^− 1^) was prepared in distilled water and stored at 4 °C in a container shielded from light. Sufficient quantity of K_2_HPO_4_ was dissolved in distilled water to prepare 0.9 g.mL^− 1^ solution. To generate the calibration curve and quality control of plasma samples, drug-free plasma was spiked with IS standard solutions. The Iranian Blood Transfusion Organization (Tabriz, Iran) provided drugs-free plasma samples, referred to as blank plasma, was kept at -20 °C until being spiked and employed in the analysis. For analysis of samples, the excitation and emission wavelengths were set to 270 nm and 403 nm, respectively. To achieve the maximum fluorescence intensity of a specific fluorophore, the excitation wavelength is selected prior to developing fluorescence analysis methodology. Consequently, the fluorophore is stimulated at that wavelength [37]. Herein, spectral diagram for IS is illustrated in Figure S1 in Supplementary Data where the fluorescence intensity of IS excitation is plotted versus the measured wavelength. Ten nm band pass was selected for both the excitation and emission beams. Fourier transform infrared spectroscopy (Bomem, Canada) was employed to observe the chemical structure of DES.

### Preparation of DES

**Fig. 1:**
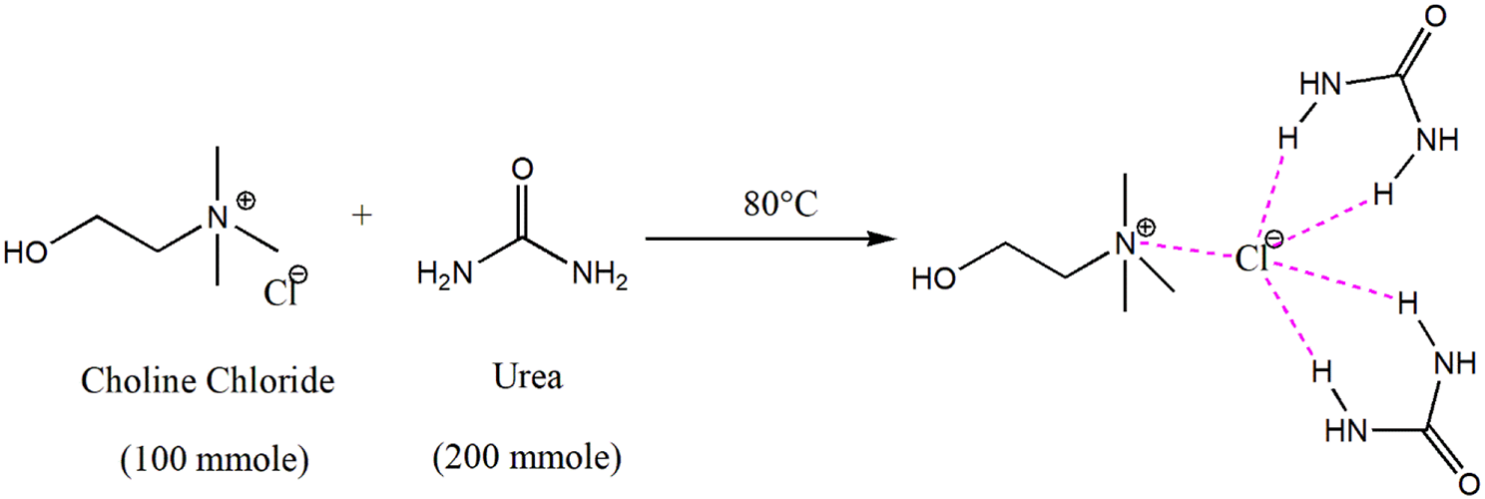
Choline chloride-urea preparation process

In this study based on the reported methods in the literature [38], ChCl-urea (1:2) was prepared as a deep eutectic mixture (Fig. 1). ChCl and urea were stirred at 80 ºC to form a colorless and consistent liquid. Fourier transform infrared spectroscopy (FT-IR) analysis was performed on individual components of DES and synthesized ChCl-urea DES as well.

### Developing and optimization method for extraction of IS by DES

A liquid-liquid extraction by DES was employed for the analysis of IS. In this study, the effect of major factors on the extraction including the speed and time of shaking, DES/salt volume ratio, salt concentration, temperature, and pH of the aqueous two-phase systems were explored for the first and back-extraction processes. The optimal extraction conditions were determined by comparing the fluorescence intensities of IS and blank.

Initially, we optimized shaking speed, shaking time, and DES/salt volume ratio for the first extraction and then considering the optimum conditions back extraction process was optimized. For this, first ChCl-urea/K_2_HPO_4_ solution (0.9 g.mL^− 1^) as the aqueous two-phase system was utilized for the extraction of IS. Then, the test tubes were centrifuged and the time and speed were optimized for the first extraction. For back extraction, the resulting supernatant was centrifuged to extract IS from the DES-rich phase to the salt-rich phase. Finally, by 2-fold diluting the salt-rich phase, the fluorescence intensity was measured. To avoid the interfering effect of sample components on fluorescence intensity, in all the experiments appropriate controls containing the same composition as the real samples but without IS were used.

In this study, the effect of separation speed on the extraction efficiency of the first extraction was also analyzed by setting only the first extraction at different shaking speeds including 4000, 6000, and 8000 rpm. In addition, the concentration of salt (0.9 g.mL^− 1^ and 1.2 g.mL^− 1^) and the volume ratios of DES to salt of 1, 1.25, and 1.5 were used for the first extraction. To examine the effect of time on extraction, the mixture was centrifuged at different shaking times of 2, 5, and 10 min.

Based on the obtained results from the first extraction optimization, the above-mentioned parameters were applied for the back-extraction process as well. Moreover, the influence of pH on the extraction of IS was examined at the pH values of 1, 7, and 13 by employing hydrochloric acid solution, distilled water and sodium hydroxide in back-extraction process.

Finally, the temperature of spectrofluorimeter was set at 5–35ºC to find the optimum value.

### Application of established method for the extraction of IS from plasma sample

**Fig. 2:**
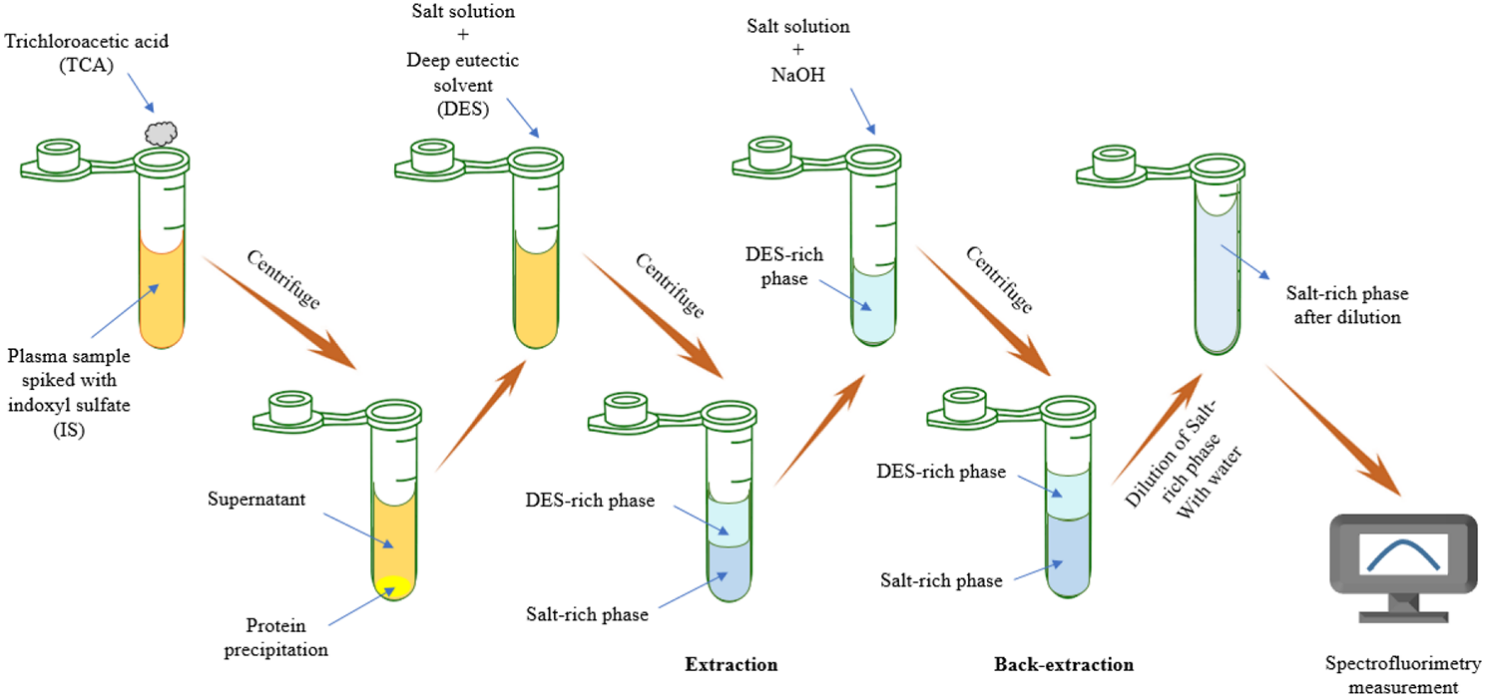
Plasma sample preparation process

One hundred mg of TCA was added to 250 µL of plasma sample which spiked with specific amount of IS to precipitate the proteins. After centrifugation for 10 min at 6000 rpm, 150 µL of clear supernatant, containing IS, was transferred in a 2 mL microtube. Then, 200 µL (0.9 g.mL^− 1^) of K_2_HPO_4_ and 250 µL of DES (choline chlorideChCl-urea 1:2) was added; and centrifuged for 2 min at 8000 rpm to form a two-phase aqueous system. Back-extraction of IS from DES-rich phase is important for removing interferences. To achieve this goal, the supernatant phase, which was a clear liquid, was transferred to another microtube. Nine hundred microliters of K_2_HPO_4_ (0.9 g.mL^− 1^) were added to DES-rich phase of previous step (400 µL) to extract IS from DES-rich phase to the salt-rich phase. This step was followed by adding 100 µL of NaOH (1 M) to the two-phase system to provide basic media. Ultimately, the fluorescence intensity of the sample was measured using a 10-mm quartz cell on a Jasco FP-750 spectrofluorometer. Each measurement was based on the mean of three replicates. Fig. 2 illustrates the plasma sample preparation process schematically.

### Calibration curve and validity of method

In a bioanalytical approach, a calibration curve is a linear relationship developed by the least squares method between concentration (the independent variable) and response (the dependent variable). This relationship is established to estimate the concentrations of analyte in a complex matrix [39].

Therefore, the calibration curve has been developed based on different IS concentrations spiked to plasma samples after extraction vs. fluorescence intensity under the ideal conditions for the applied analytical method.

Validation of all proposed analytical techniques is necessary to confirm that routine measurements of the analyte in samples are within a reasonable range of the true values [40]. Validation of the primary parameters, such as linearity, accuracy, precision, specificity, selectivity, sensitivity, and stability testing, is required [39]. In the following sections validation parameters are discussed.

### Selectivity

In order to measure the changes in fluorescence intensity (ΔF%), the analytical method’s selectivity was estimated in the presence of commonly administered medications in the plasma of patients with chronic kidney disease (CKD) and some important physiologic cations. The concentration of each drug/cation was examined at 3-fold above the maximum plasma concentration level. Moreover, the selectivity of established method was evaluated in three plasma samples from different sources.

### Real plasma sample preparation

Real plasma samples were collected from patients with end-stage renal disease (ESRD) in Sina Hospital, Tabriz University of Medical Sciences, Tabriz, Iran. The experiment was approved by the Tabriz University of Medical Sciences Ethics Committee (code: IR.TBZMED.REC.1402.154) and all sample donors completed a written consent form. The separation of plasma from blood samples (8 mL) occurred through centrifugation. Then, plasma was separated and stored at -70 °C until analysis.

## Results and discussion

### FT-IR results of DES

FTIR spectroscopy was applied to study the interface between distinct functional groups of the compounds (ChCl/urea) and identify the construction of hydrogen bond. Figure S2 in Supplementary Data shows the FTIR spectra of ChCl, urea, and ChCl/urea (1:2). Moreover, spectra resulted from a KBr disc of urea and ChCl. The most important IR absorption regions of urea include a double peak of N-H stretching vibrations at 3200–3600 cm^− 1^, carbonyl group (C = O) absorption at 1465 cm^− 1^. The signal at 1157 cm^− 1^ is also associated with C-N vibrations, and the absorption peak at 1500 to 400 cm^− 1^ is the indicator of the fingerprint region for the identification of urea. For pure and dried ChCl the vibration at 3247 cm^− 1^ corresponds to –OH stretching and the signals at 2954 cm^− 1^ and 2900 cm^− 1^ are attributed to N-H stretching. The signals at 948 cm^− 1^ and 1481 cm^− 1^ are related to C-N group and CH_2_ bending of ChCl, respectively. In addition, the wide signal of –OH group can be observed in the range of 3679 –3031 cm^− 1^. Finally, the band 871 cm^− 1^ is assigned to C-C stretching.

The absorption peaks at 3200–3600 cm^− 1^ in urea and 3247, 2954, and 2900 cm^–1^ in ChCl changed to a broad-ranging peak of –OH and -NH in ChCl/urea as the DES system. It occurs through the hydrogen bond formation between urea and ChCl (Cl group of ChCl and NH [41].

Main factors of extraction procedures including the DES/salt volume ratio, the concentration of salt (K_2_HPO_4_), and centrifuging time and speed were optimized. In addition, in the back-extraction step, the effect of pH and temperature on fluorescence intensity of IS were checked. Under the optimized conditions for the proposed analytical method, the calibration curve was developed using different concentrations of IS spiked into plasma samples vs. fluorescence intensity, and the validity of the method was evaluated.

Finally, it was applied for quantification of IS in real samples. The results are presented in the following sections:

### Optimization of extraction process of IS

#### Effect of DES/salt (K_2_HPO_4_) volume ratio

**Fig. 3:**
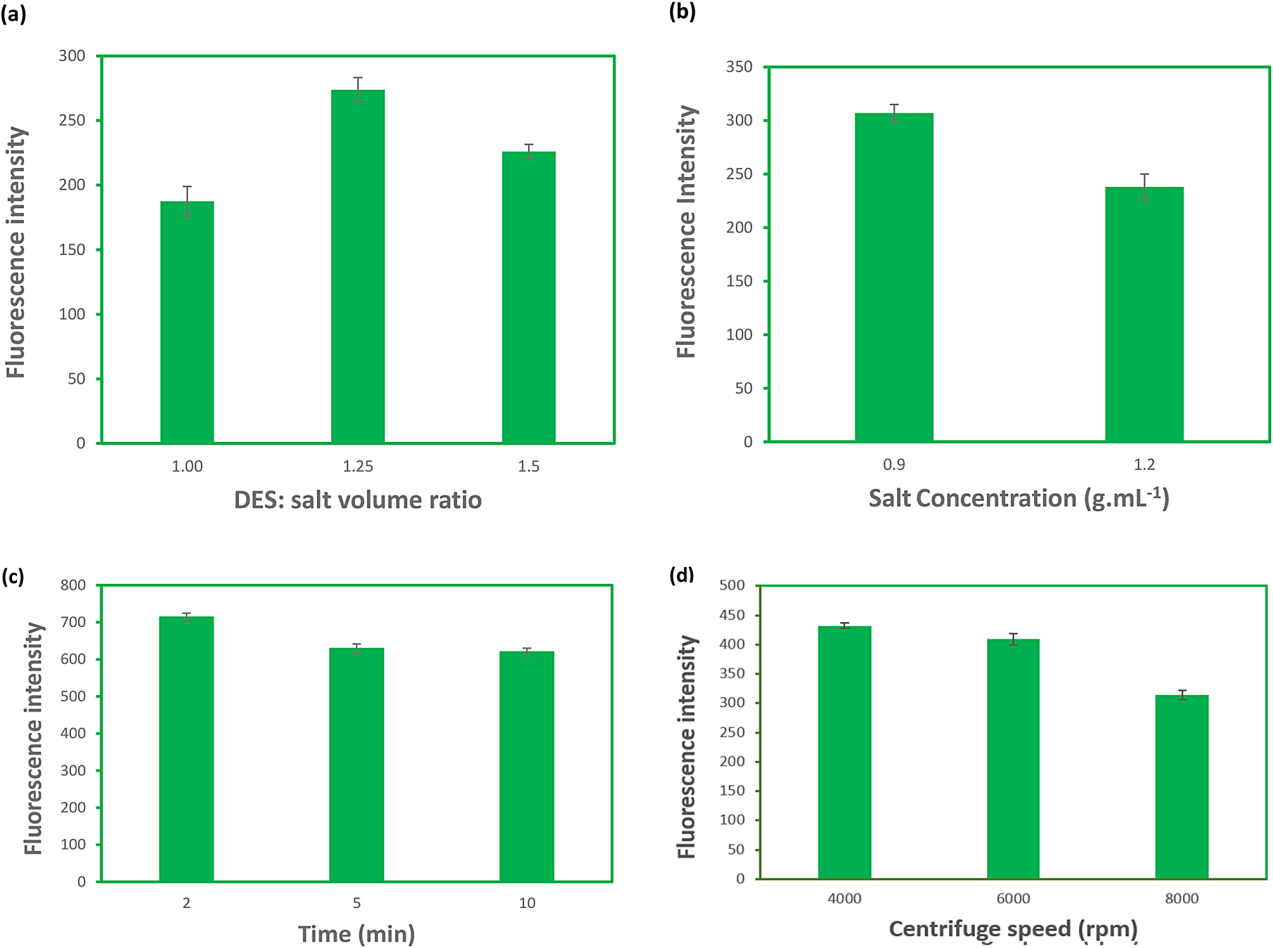
Optimization of extraction process of IS. (**a**) DES/salt volume ratio, (**b**) salt concentration, (**c**) centrifuging time, (**d**) centrifuging speed. Concentration of spiked IS: 20 µg.mL^− 1^; other experimental conditions in each step were constant (DES/salt volume ratio = 1.25, salt concentration = 0.9 g.mL^− 1^, centrifuging time = 10 min, centrifuging speed = 6000, and for back-extraction process, salt concentration = 0.9 g.mL^− 1^, centrifuging time = 10 min, centrifuging speed = 6000 rpm and pH = 13)

Effect of DES/salt (K_2_HPO_4_) volume ratio was illustrated in Fig. 3a. It shows the fluorescence intensity increased when the DES/salt volume ratio varied from 1 to 1.25, and then it decreased with further increase in K_2_HPO_4_ amount. The explanation for this change was that as a result of higher amount of DES that moderately increased, the more IS could be extracted by the DES-rich-phase. Meanwhile, with the further advance of DES amount, the fluorescence decreased due to the less solvent capacity that prevented IS transferring from salt-rich-phase into the DES-rich-phase. The optimum amount of DES/salt (K_2_HPO_4_) volume ratio was 1.25 and this ratio was adopted in the subsequent experiments.

#### Effect of salt (K_2_HPO_4_) concentration

Effect of salt (K_2_HPO_4_) concentration on fluorescence intensity was illustrated in Fig. 3b. It was demonstrated that the fluorescence intensity decreased with an increasing concentration of K_2_HPO_4_ solution from 0.9 to 1.2 g.mL^− 1^ during the extraction process.

#### Effect of centrifuging time

The effect of centrifuging time on fluorescence intensity was examined. The test tubes were centrifuged at constant rpm (6000 rpm) with various times (2, 5, and 10 min). According to the results shown in Fig. 3c, the fluorescence intensity of IS decreased as the centrifuging time increased. Finally, within 2 min for the first extraction, the fluorescence intensity reached its highest value. Therefore, 2 min was selected as the suitable shaking time for the first extraction. The important benefits of this method include shorter extraction time.

#### Effect of centrifuging speed

The effect of centrifugation speed on extraction efficiency was also analyzed. The centrifuge speed was set at different centrifuging speed including 4000, 6000, and 8000 rpm for 10 min. In light of Fig. 3d, with the increase of centrifuging time in the extraction process, the fluorescence intensity of IS also increased within 8000 rpm. Therefore, it was chosen as the proper centrifuging speed for the extraction of IS.

### Optimization of the back-extraction process of IS

#### Effect of salt (K_2_HPO_4_) concentration

**Fig. 4:**
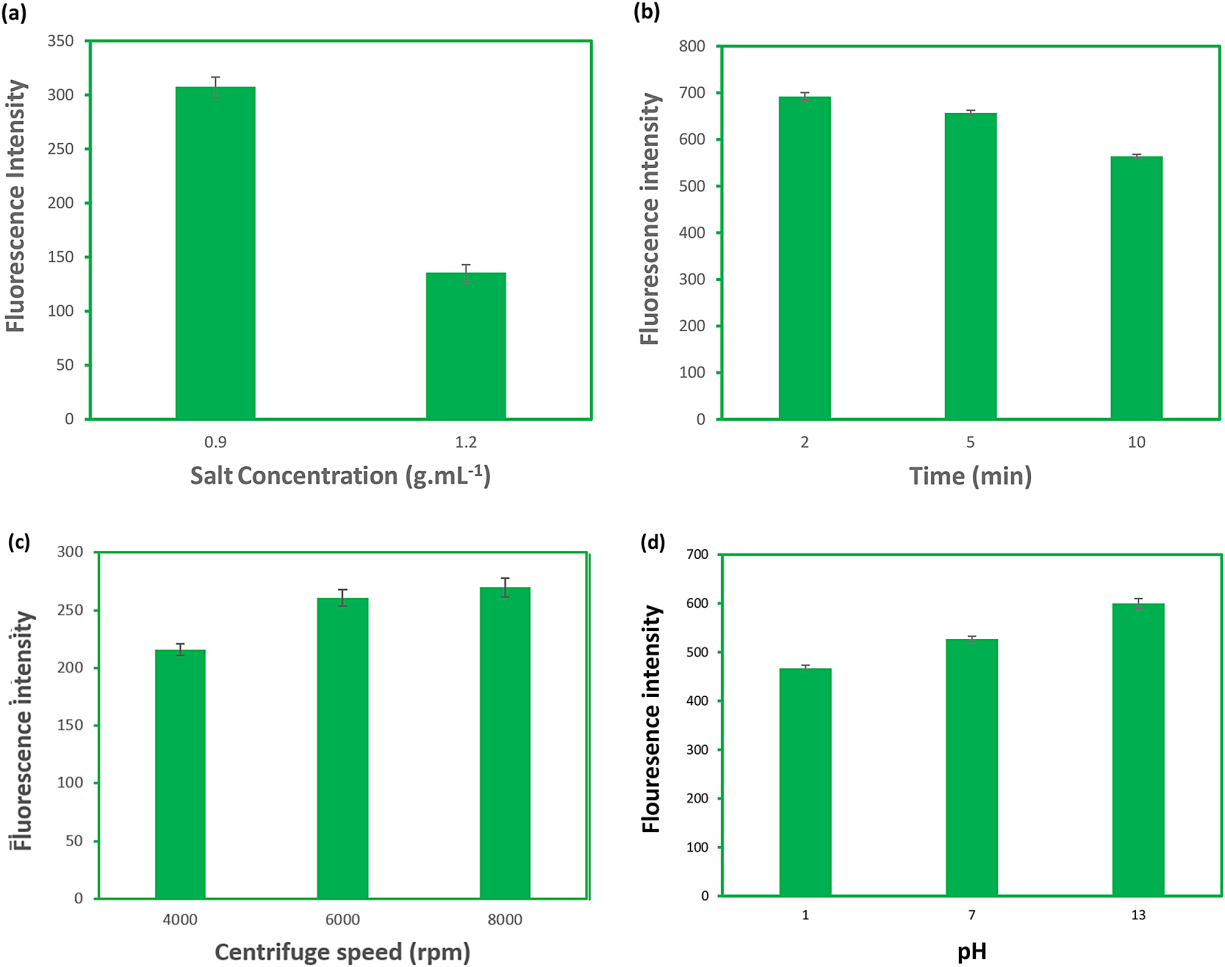
Optimization of back-extraction process of IS, (**a**) salt concentration, (**b**) centrifuging time, (**c**) centrifuging speed (**d**) pH. Concentration of spiked IS: 20 µg.mL^− 1^; other experimental conditions in each step were constant (for extraction process, DES/salt volume ratio = 1.25, salt concentration = 0.9 g.mL ^− 1^, centrifuging time = 2, centrifuging speed = 8000 and for back-extraction process, salt concentration = 0.9 g.mL^− 1^, centrifuging time = 10 min, centrifuging speed = 6000 and pH = 13)

To investigate the effect of salt concentration on the fluorescence intensity of extracted IS, the concentration of K_2_HPO_4_ was adjusted at 0.9 g.mL^− 1^ and 1.2 g.mL^− 1^ and other conditions were constant in both extraction and back-extraction processes. The fluorescence intensity of IS started to decrease when the K_2_HPO_4_ solution was higher than 0.9 g.mL^− 1^ i.e., 1.2 g.mL^− 1^). The results were illustrated in Fig. 4a. Consequently, for back-extractions, the concentration of K_2_HPO_4_ solution was identified to be 0.9 g.mL^− 1,^ as well.

#### Effect of centrifuging time

To achieve suitable phase separation time and the optimum extraction efficiency for back-extraction process, the test tubes were centrifuged with different periods of 2, 5, and 10 min. The highest fluorescence intensity yielded within 2 min according to Fig. 4b.

#### Effect of centrifuging speed

In order to attain the most suitable condition for shaking speed in back-extraction efficiency, we examined 4000, 6000, and 8000 rpm under unchanged conditions. The fluorescence intensity was at its highest value within shaking speed of 4000 rpm (Fig. 4c). Hence, 4000 rpm was selected as the optimum shaking speed for the back-extractions.

#### Effect of the pH value

The influence of pH on the back-extraction of IS was examined within the pH range of 1 to 13 by employing hydrochloric acid (1 M), water, and sodium hydroxide (1 M) in back-extraction process. The greatest value of fluorescence intensity has been attained at pH 13, as illustrated in Fig. 4d. As a result, pH 13 was determined for additional studies. It was could be related to deprotonation in basic media resulted in the formation of negative charges [42]. It implied that the ionization amount might be a major factor in the extraction of IS in the pH range of the aqueous two-phase system of DES/ K_2_HPO_4_.

#### Effect of the temperature

The rise in temperature causes more molecular collisions and a drop in fluorescence intensity, whereas a reduction in temperature causes fewer molecular collisions and more fluorescence intensity [43]. The fluorescence intensity decreased from 686 to 339 in the 5–35 ºC range. Due to the high fluorescence intensity, the ideal temperature was determined to be 5 ºC for further studies.

### Validation of the developed analytical method

The established method for extraction of IS were validated under the optimized conditions and the results have been reported as follow:

#### Calibration curve and linearity

After optimization, a linear correlation was detected between the fluorescence intensity and IS concentrations within the 20–160 µg.mL^− 1^ range, with a determination coefficient (R^2^) of 0.99.

**Fig. 5:**
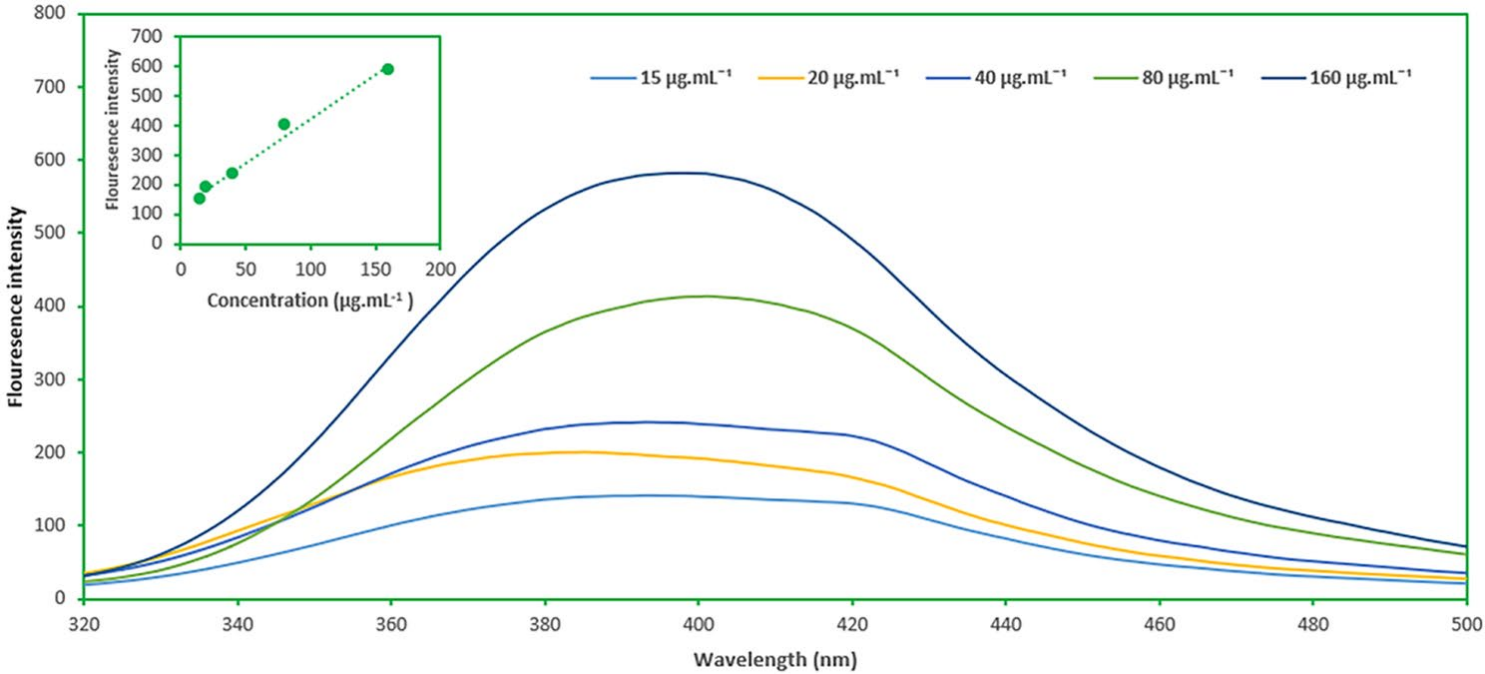
Calibration curve and fluorescence spectra of adding different concentrations of IS

Fig. 5 shows the calibration curve and fluorescence spectra of adding different concentration of IS through the proposed method. The relative standard deviation (RSD) of each concentration point on the curve is less than 20%. Based on the obtained results, the lower limit of quantification (LLOQ) of the method was 20 µg.mL^− 1^, because of the RSD value and back-calculated error of this point was less than 20%. Lower of detection (LOD) of the proposed method was 6.1 µg.mL^− 1^ based on the Eq. 3 S/b, where S is the standard deviation and b is the slope of the calibration plot.

#### Accuracy and precision

Three different levels of IS as quality control (QC) concentrations were added to plasma samples. The QC samples were evaluated in three consecutive days at three different concentrations (35, 70, and 110 µg.mL^− 1^) in order to ascertain the precision and accuracy of IS concentration determination in plasma. The results are summarized in Table 2. The mean relative recovery and RSD values for all data points for IS were 89.7% and 13.6%, respectively. The obtained findings for the accuracy (relative recovery) were between 80 and 96% and the precision, i.e., %RSD values, were less than 20% at all examined concentrations.

**Table 2:** Recoveries for extraction and determination of IS in spiked plasma samples with spectrofluorimetry

Nominal concentration (µg.mL^− 1^, *n* = 3)	Found concentration (µg.mL^− 1^±SD, *n* = 3)	RSD%	Recovery%
Intra-day			
35	30 ± 2.3	7.8	85
70	68 ± 10.2	15.0	96
110	96 ± 6.0	6.3	88
**Inter-day**			
35	32 ± 4.2	13.0	91
70	66 ± 6.9	10.5	93
110	93 ± 7.5	8.1	85

#### Stability

The stability of a drug in biofluids is affected by the matrix, the container system, the drug’s chemical characteristics, and the storage environments. Stability experiment conditions should resemble scenarios that are likely to appear during the processing and analysis of real samples. A set of samples made from a newly made stock solution of the analyte in the proper biological matrix that is interference-free and free of analytes should be used for all stability measurements [44]. Herein, the stability results are summarized in Table 3 where the highest deviation was obtained for 20 µg.mL^− 1^ of IS at room temperature (RE % = 19.1%). These values are acceptable (< 20%) for biological samples according to validation guidelines in biological samples [45].

**Table 3:** Stability data for quantification of IS based on the established method in human plasma samples

Concentration (µg.mL^− 1^)	Room temperature stability	Freeze–thaw stability
	ΔF%	ΔF%
35	13	9
70	8	7
110	3	3

### Selectivity

In order to measure the changes in fluorescence intensity (ΔF%), the analytical method’s selectivity was estimated in the presence of co-administered medications in the plasma of patients CKD. Three-fold above the maximum concentration (Cmax) of each drug was examined. Table 4 summarized the influence of these medications on IS fluorescence intensity of IS (20 µg.mL^− 1^). The ΔF% were limited to less than 13% at maximum. Some of the studied medications in Table 4 exhibit high intrinsic fluorescence. Nevertheless, no significant change in the fluorescence intensity of IS in the presence of the examined compounds was observed, which is due to the extraction process, the small amount of sample and its dilution, and the difference between the maximal excitation and emission wavelengths of IS and those of the co-administered medications [27]. Additionally, various plasma samples from three healthy individuals were spiked with the same concentration of IS (20 µg.mL^− 1^) to assess the effects of matrices. With a ΔF% of less than 15%, the data had no noticeable effect on the developed method for the determination of IS. These results indicated an adequate selectivity for the proposed method of IS assay in plasma samples of ESRD patients on disease related medications.

**Table 4:** The selectivity of developed method in presence co-administered medications in ESRD patients on the fluorescence intensity of IS (20 µg.mL^− 1^)

Drug	Maximum plasma concentration (µg.mL^− 1^)	Reference	ΔF%
Ca ^2+^	2.5	[69]	12
K^+^	5.0	[70]	10
Na^+^	142	[70]	9
Mn^2+^	4.3 × 10^− 3^	[71]	8
Zn^2+^	111.30	[72]	13
Cu^2+^	4.3 × 10^− 3^	[73]	8
Losartan	1.2	[74]	12
Allopurinol	20	[74]	8
Atorvastatin	12.08	[74]	12
Vitamin E	17	[74]	9
Captopril	0.5	[74]	9
Furosemide	6	[74]	10

### Employing the established technique on real samples

In order to assess the capability of the currently employed protocol, real plasma samples were used for measuring the IS level based on the established method in this study. Blood samples were afforded from 2 individuals suffering from ESRD at Sina Hospital in Tabriz, Iran. The blood samples were centrifuged at 2,000 rpm for 10 min after to avoid the coagulation [46]. Prior to the analysis, the obtained serum aliquots were kept at -70 °C. For the analysis, 250 µL of the plasma samples were subjected to the extraction based on the proposed method, and then the fluorescence quantification of IS was performed. The results are presented in Table 5. All of the obtained concentration in the patients’ plasma samples were at the linear range of the calibration curve. The concentration of IS in the studied ESRD patients is greater than 40 µg.mL^− 1^ which is in agreement with the reported concentrations of IS in CKD patients [47], and it is further validation for the accuracy of developed method. Moreover, standard addition method was employed on real samples to determine the accuracy of the developed protocol for the IS measurement [48]. The obtained recovery was between 98 and 117% and it indicates acceptable accuracy of the established method.

**Table 5:** Determination of IS in real plasma samples by developed method and evaluation the accuracy of the method by standard addition method

Sample	Fluorescence intensity	Concentration (µg.mL^− 1^)	Recovery%
**A**	146	46	-
**A + 30 (µg.mL** ^**− 1**^ **)**	234	76	100
**A + 80 (µg.mL** ^**− 1**^ **)**	400	134	110
**B**	189	61	-
**B + 30 (µg.mL** ^**− 1**^ **)**	291	96	117
**B + 80 (µg.mL** ^**− 1**^ **)**	470	158	98

## Comparison with other methods (advantages and disadvantages and limitations)

Numerous approaches have been developed to assess the IS level in biological fluids including chromatographic methods like chromatography (GC) and liquid chromatography (LC) and non-chromatographic methods such as electrochemical [49, 50], enzymatic [51], and spectroscopic [52] techniques. LC methods with mass (MS) or MS/MS [53–64] detectors are common for the analysis of IS in biological fluids. However, they are time-consuming, laborious, and need specialized and expensive equipment. Although, some of these methods especially chromatography methods offer high sensitivity (Table 1) and effective separation, they are not free from disadvantages. Therefore, the necessity of developing a simple, rapid, and cost-effective technique based on quantification by rapid methods like fluorescence with a reliable clean-up process to remove matrix effect through environmentally friendly solvents (DES) is crucial. Generally, the sensitivity of established method is enough to quantify IS in real plasma samples of CKD patients.

## Conclusion

A new extraction methodology based on DES and spectrofluorimetry was established to extract and analyze IS concentration in human plasma samples. The method was applied for quantification of IS in ESRD patients. The use of DES extraction solvent instead of high toxicity organic solvents is environmentally beneficial. The two-step proposed extraction method has an ideal clean-up result and is capable of eliminating the plasma matrices effect. Due to the simplicity, cost-effectiveness, and rapidness of the extraction technique, it is applicable for removing the effects of complex matrices like plasma. Hence, spectrofluorimetric determination through extraction can be effectively implemented as a typical analytical strategy for detecting IS since it provides good precision, selectivity, and stability.

### Electronic supplementary material

Below is the link to the electronic supplementary material.

Supplementary Material 1

## Data Availability

Data generated or analyzed during this study are available from the corresponding author upon reasonable request.
