# Supplementary material for: Determination of indoxyl sulfate by spectrofluorimetric method in human plasma through extraction with deep eutectic solvent
Source: BMC Chem. 2024 Mar 30;18(1):61. doi: 10.1186/s13065-024-01172-9 (PMC10981813; doi:10.1186/s13065-024-01172-9)
Supplement: Supplementary file 1 — Supplementary Material 1 [file 13065_2024_1172_MOESM1_ESM.docx]

**Supplementary Data**

# Figure S1. IS excitation and emission spectra

**Figure S2.** FT-IR analysis of choline chloride (ChCl), urea and DES.
